# Enhancing the Antibiotic Antibacterial Effect by Sub Lethal Tellurite Concentrations: Tellurite and Cefotaxime Act Synergistically in *Escherichia coli*

**DOI:** 10.1371/journal.pone.0035452

**Published:** 2012-04-20

**Authors:** Roberto C. Molina-Quiroz, Claudia M. Muñoz-Villagrán, Erick de la Torre, Juan C. Tantaleán, Claudio C. Vásquez, José M. Pérez-Donoso

**Affiliations:** 1 Laboratorio de Microbiología Molecular, Departamento de Biología, Facultad de Química y Biología, Universidad de Santiago de Chile, Santiago de Chile, Chile; 2 Laboratorio de Microbiología y Biotecnología, Facultad de Ciencias, Universidad San Luis Gonzaga, Ica, Perú; Auburn University, United States of America

## Abstract

The emergence of antibiotic-resistant pathogenic bacteria during the last decades has become a public health concern worldwide. Aiming to explore new alternatives to treat antibiotic-resistant bacteria and given that the tellurium oxyanion tellurite is highly toxic for most microorganisms, we evaluated the ability of sub lethal tellurite concentrations to strengthen the effect of several antibiotics. Tellurite, at nM or µM concentrations, increased importantly the toxicity of defined antibacterials. This was observed with both Gram negative and Gram positive bacteria, irrespective of the antibiotic or tellurite tolerance of the particular microorganism. The tellurite-mediated antibiotic-potentiating effect occurs in laboratory and clinical, uropathogenic *Escherichia coli*, especially with antibiotics disturbing the cell wall (ampicillin, cefotaxime) or protein synthesis (tetracycline, chloramphenicol, gentamicin). In particular, the effect of tellurite on the activity of the clinically-relevant, third-generation cephalosporin (cefotaxime), was evaluated. Cell viability assays showed that tellurite and cefotaxime act synergistically against *E. coli*. In conclusion, using tellurite like an adjuvant could be of great help to cope with several multi-resistant pathogens.

## Introduction

The constant emergence of clinically-relevant pathogens exhibiting high levels of antibiotic resistance is nowadays a worldwide health problem that poses new challenges to the scientific community. Such scenario is even more worrying given the ability of some pathogens to use antibiotics as the sole carbon source [1].

During the last 50 years, the pharmaceutical industry has introduced only one new antibiotic into the market. However and even if new compounds with antibiotic ability are discovered, the emergence of resistant strains is only a matter of time. This is the main reason to look for new treatments, and in this context the use of compounds strengthening the antibiotic effect is a choice that worth to be evaluated [2]. Recently, it has been reported that some antibiotics can act in a synergistic manner when used in conjunction with genetically-modified bacteriophages or organometallic compounds, among others [3], [4], [5].

In 2007, Kohanski *et al.* [6] showed that a common mechanism underlying the toxicity of bactericidal antibiotics involves the generation of the highly reactive oxygen species (ROS), hydroxyl radical. Overall, this observation evidences that the general mechanism(s) underlying antibiotic-toxicity are not fully understood to date. On the other hand, in 1932 Fleming reported the antibacterial properties of tellurite (TeO_3_^2−^) and penicillin [7] and since then TeO_3_^2−^ has been used routinely to isolate tellurite-resistant strains as *Escherichia coli* O157, *Proteus spp.*, and other bacteria [8].

During the last years our group has been interested in studying the underlying molecular mechanism(s) of tellurite toxicity. It has been shown that part of it results from ROS generation [9], damage to metabolic enzymes [10], [11], glutathione depletion [12] or lipid peroxidation [13]. In this context, based on the high toxicity exhibited by TeO_3_^2−^ against bacteria, its numerous cell targets [14] and its apparent low noxiousness to eukaryotic cells [15], we hypothesized that TeO_3_^2−^ could increase significantly the antimicrobial effect of antibiotics.

In this work we report that sub lethal tellurite concentrations increase the effect of ampicillin, tetracycline, chloramphenicol or cefotaxime against *E. coli* and *Pseudomonas aeruginosa*. A similar, but reduced effect was observed with the highly tellurite- and antibiotic-resistant *Staphylococcus aureus*. Especially interesting was the effect with cefotaxime, a widely-used, third-generation cephalosporin, which was found to act synergistically with tellurite against *E. coli*.

## Results

The ability of non-lethal tellurite concentrations to increase the antibacterial effect was assessed by determining growth inhibition zones. Antibiotics targeting different cellular processes were tested in the absence or presence of sub lethal tellurite concentrations.

**Figure 1:**
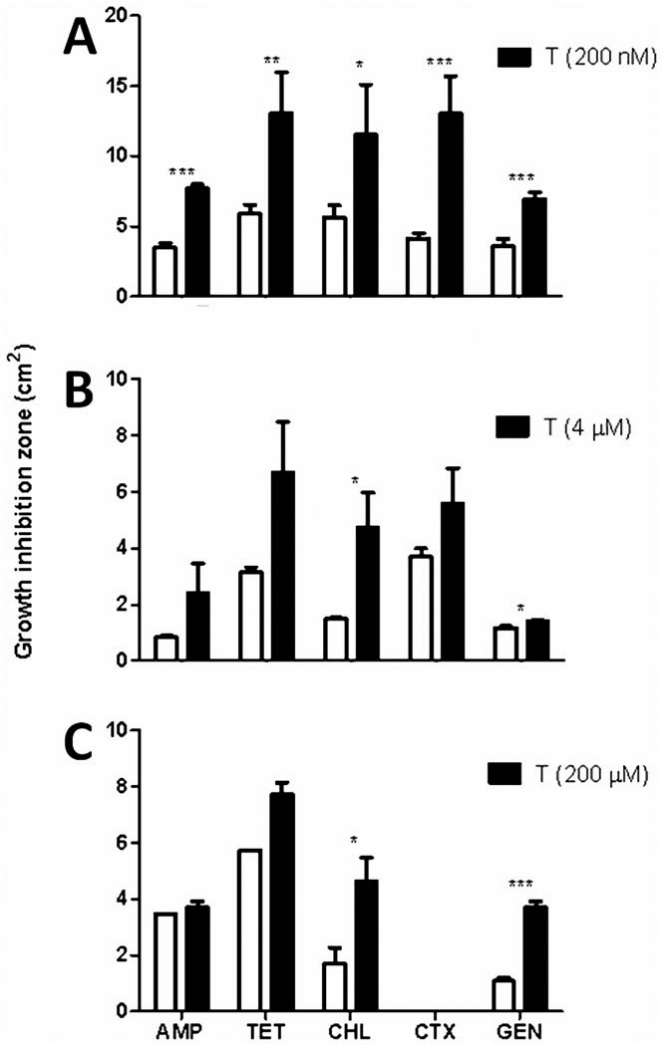
Tellurite-mediated antibiotic-potentiating effect in different bacteria. Antibiotic-mediated inhibition growth zones were determined for *E. coli* (A), *P. aeruginosa* (B) and *S. aureus* (C) grown in the absence (white bars) or presence of the indicated tellurite (T) concentrations as described in Methods. Values represent the average of at least 4 independent trials and significance was determined using t-test analysis (p<0.05). Significance values are (*) p<0.05, (**) p<0.01 and (***) p<0.001.

Bacterial species displaying distinct susceptibility to antibiotics and TeO_3_^2−^ were evaluated to determine if the potentiating effect was also observed with tellurite- or antibiotic-resistant bacteria. Tellurite-mediated antibiotic-potentiating effects were observed with AMP, CHL, TET, GEN and CTX when *E. coli* (highly sensitive to tellurite, MIC 4 µM) was grown in tellurite-amended LB plates. Approximately a 3-fold increase in growth inhibition zones was observed with TET, CHL and CTX (Fig. 1A). In turn, when *P. aeruginosa* was exposed to different antibiotics in the presence of 4 µM TeO_3_^2−^ (MIC/80), a significant potentiating-effect was observed only with CHL and GEN (Fig. 1B). Similar results were obtained with *S. aureus* grown in 200 µM tellurite (MIC/20)-containing plates (Fig. 1C).

Differences in growth inhibition areas observed among these bacterial species are most probably due to their different susceptibility to tellurite and antibiotics. *P. aeruginosa* exhibited smaller inhibition zones than *E. coli*, which may reflect antibiotic resistance genes that are absent in *E. coli* K12-derived laboratory strains [16]. In our hands and depending on the particular antibiotic, MIC values for these antibacterials decreased 25–75% in the presence of sublethal tellurite concentrations.

Particularly interesting was the effect in *E. coli* exposed to CTX, where the most significant inhibition zone increase was observed in the presence of tellurite (Fig. 1A). CTX, a third-generation cephalosporin, is routinely used to treat infections caused by Gram-negative and Gram-positive pathogens and also as prophylactic strategy [17]. Given the effect observed in sensitivity to CTX and its clinical relevance, the tellurite-dependent potentiation on CTX effect was further explored.

**Figure 2:**
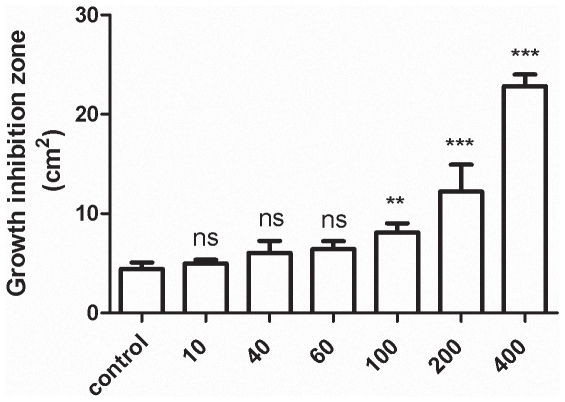
Minimal tellurite concentration causing a cefotaxime-potentiating effect in *E. coli*. Inhibition growth zones were determined as described in Methods using LB plates amended with the indicated sub lethal tellurite concentrations (nM).

The minimal concentration of tellurite displaying CTX potentiation was determined. A dose-dependent effect was observed when tellurite concentrations ranging from 1/10 up to 1/1,000 of *E. coli* MIC were evaluated (Fig. 2). Although the maximal effect was observed at 400 nM, half of this concentration was used since the potentiating effect was still significant and because this concentration seems not to affect eukaryotic cells [15], [18], [19].

The cefotaxime MIC for *E. coli* was diminished 4 fold (0.13 to 0.03 µg/ml) when grown in the presence of tellurite. Surprisingly, the CTX MIC for the antibiotic-resistant bacteria *P. aeruginosa* decreased >30 fold (300 to 9.3 µg/ml) in the presence of 4 µM tellurite. Since the CTX MIC is the same for pathogenic [20] and laboratory *E. coli* strains, these results could be important in terms of future applications of tellurite-mediated cefotaxime potentiation. In this context and aiming to assess if the tellurite-potentiating antibiotic effect was also observed with pathogenic bacteria, clinical isolates were exposed to both antibacterials. Growth inhibition zones resulting from antibiotic exposure in the presence or absence of 200 or 400 nM tellurite were determined for 20 clinical coliform isolates from patients suffering urinary infection. A dose-dependent, tellurite-potentiating effect was observed with all tested antibiotics. Interestingly, the most robust effect was again observed with CTX, which was over 2 fold than that observed with other antibiotics as STR, AMK, KAN and TOB (Table 1).

**Table 1:** Tellurite-mediated antibiotic-potentiating effect in clinical isolates.

Antibacterial		Tellurite (nM)	
	0	200	400
Ctx	2.29	3.55 (55)	4.60 (100)
Cefl	1.31	1.87 (43)	2.39 (83)
Amp	1.50	2.07 (38)	2.31 (54)
Neo	1.45	2.11 (45)	2.63 (81)
Str	2.32	2.90 (24)	3.52 (51)
Gen	2.11	2.78 (32)	3.17 (51)
Amk	2.18	2.68 (22)	3.16 (45)
Kan	2.09	2.58 (23)	3.45 (65)
Tob	4.49	4.90 (9)	5.91 (31)

Antibiotic susceptibility, in the absence or presence of the indicated tellurite concentrations, was assessed by growth inhibition zones (cm^2^) as described in Methods. Parentheses indicate per cent of susceptibility increase regarding the respective control.

**Figure 3:**
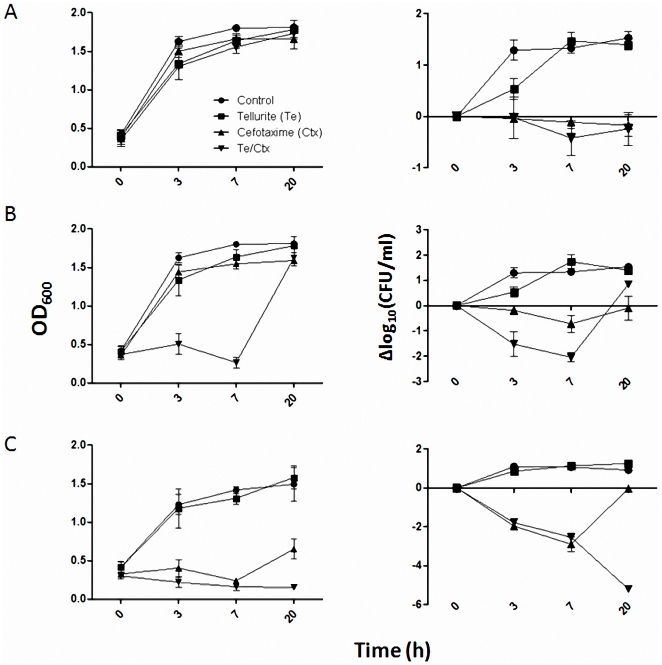
Cefotaxime and potassium tellurite acts synergistically in *E. coli*. Growth curves (left panels) and cell viability (right panels) were determined at the indicated time intervals for *E. coli* exposed to 0.065 (A, sublethal), 0.13 (B, MIC) and 0.5 µg/ml (C, lethal) CTX in the absence or presence of 200 nM tellurite. Controls contained no tellurite or cefotaxime. Data represent the mean of at least 3 independent trials. Refer to inset in panel A for symbol meaning.

To characterize the type of antimicrobial effect after exposing bacteria simultaneously to tellurite and CTX, cell viability determinations were carried out using different antibiotic concentrations in the presence of the tellurium oxyanion (Fig. 3). Growth and cell viability were not severely affected when *E. coli* was exposed to 200 nM tellurite. In fact, normal growth and viability was restored after 3 h exposure (Fig. 3, squares).

While growth was not affected when cells were exposed concurrently to 0.065 µg/ml CTX (MIC 0.13 µg/ml) and tellurite (200 nM), the number of viable cells was strongly decreased as determined by CFU counting (Fig. 3A). A similar result was obtained upon exposing to the antibiotic alone, suggesting that the effect observed with both CTX+tellurite depends mainly on antibiotic-mediated damage.

When cells were grown in the presence of 0.13 µg/ml cefotaxime, growth and viability recovered only after 7 h treatment. The observed potentiating-effect at 3 or 7 h exposure cannot be explained as the sum of tellurite- and CTX-independent effects (Fig. 3B). This indicates a tellurite/cefotaxime-mediated synergistic effect in *E. coli*.

Finally, when the potentiating effect was assessed in cells exposed to lethal CTX concentrations (0.5 µg/ml), the synergy was represented by a difference of ∼5 Δlog_10_ units after 20 h and growth or cell viability was not recovered at all (Fig. 3C).

## Discussion

Bacterial multi-resistance to different antibiotics has become a severe problem worldwide. To face this situation, the scientific community and pharmaceutical industry have made important efforts to discover new compounds exhibiting antibacterial properties. However, in the last 40 years these efforts have resulted in the discovery of only 2 new antibiotics, the oxazolidinone linezolid and the lipopeptide daptomycin [21], [22].

The conventional treatment of bacterial infections currently lies in administering antibiotics alone or in combination [23], or using last-generation antibiotics as the case of the multi-resistant *Enterobacteriaceae* with carbapenems [24]. In spite of this, strains resistant to these new antibacterials emerge continuously, making the situation critical.

Horizontal gene transfer is the principal mode of acquiring new information by bacteria thus allowing them to cope with new antibacterial agents. In this context, the idea of using 2 different antibiotics to treat bacterial infections seems reasonable but there is still a risk of acquiring resistance determinants. To avoid multi-resistance emergence, the use of compounds exhibiting multi-target toxicity is an interesting and novel alternative, since getting a mutation or acquiring genetic determinants against these new compounds is minimal. In this context, using molecules as tellurite to potentiate the antibacterial effect seems to be a fine approach. Although information regarding TeO_3_^2−^ toxicity for eukaryotic cells is scarce to date, it has been shown that 50 µM tellurite (>125-fold the maximal dose used in this work) seems not to affect the viability of eukaryotic cells [15]. In fact, in different cell lines death occurs at ∼160–1,600 µM tellurite, as compared to the *E. coli* 4 µM killing-dose. Despite the important effect in survival observed in neurons [19] and erythrocytes exposed to 100–500 µM tellurite [25], no significant effects have been reported when lower concentrations were used [26]. Indeed, a therapeutic use of tellurite as a red cell antisickling agent has been proposed [24]. Although rats receiving 8 µM tellurite daily doses did not reveal toxic effects over a year, tellurite-treated animals showed increased mortality after 19 months [27], [28]. Despite these considerations, it results obvious that the real effect of tellurite on eukaryotic cells has not been well established to date.

Considering the use of tellurite as strategy to kill bacteria without affecting eukaryotic host cells, it was determined that sub lethal TeO_3_^2−^concentrations increase the susceptibility of different bacterial species to various antibiotics in either LB or Müeller-Hinton media (not shown). The fact that increased growth inhibition zones were observed with most tested antibiotics in the presence of TeO_3_^2−^ (Fig. 1) suggests that this condition may not be related to the antibiotic's specific target. This allows hypothesizing a common mechanism underlying the observed potentiating effect. As shown by Kohanski et al. for bactericidal antibiotics [6] and by our group for tellurite [9], these compounds promote oxidative stress which could in part explain their toxicity. Experiments to address this issue are under way in our laboratory.

Tellurite-mediated potentiation of TET, GEN and CHL is probably consequence of a combined effect upon protein synthesis (mediated by the antibiotic) and tellurite-induced protein oxidation. In this context, *E. coli* protein misfolding/mistranslation or oxidation has been observed upon exposure to some aminoglycosides [29] or tellurite [9], respectively.

Major changes in growth inhibition zones observed with Gram negative bacteria facing simultaneously tellurite and antibiotics are probably consequence of their high tellurite susceptibility as compared to that exhibited by Gram positive microorganisms [14]. Differences in growth inhibition areas between *E. coli* and *P. aeruginosa* may be explained because of the high antibiotic-resistance levels exhibited by the last bacterium. In spite of this, its susceptibility to antibiotics can be increased in the presence of low tellurite concentrations (Fig. 1B). On the other hand, a less robust effect was observed when *S. aureus* was exposed to tellurite and antibiotics, probably because the high resistance to both toxicants exhibited by this Gram positive rod.

A synergistic effect, evidenced by a difference of >2 log units in cell viability, was observed when characterizing the magnitude and the type of tellurite-mediated CTX-potentiating effect (Figs. 3B and C). This was also the case with growth curves, where an important decrease in OD_600_ was observed when exposing to both antimicrobials (Fig. 3). Although viability was rather unaltered, increased turbidity was observed when *E. coli* was exposed to 0.13 µg/ml CTX (Fig. 3B), a result that might be explained by cell filamentation upon exposition to β-lactam agents as cefotaxime [30]. Cell viability was recovered only 7 h after exposing to a lethal cefotaxime concentration (0.5 µg/ml) (Fig. 3C), a result that may reflect a decreased antibiotic bioavailability because of covalent linkage formation with bacterial penicillin binding proteins (PBPs), as has been described for other β-lactam antibiotics [31].

Since hydroxyl radical and superoxide formation occurs during *E. coli* exposure to tellurite or bactericidal antibiotics, respectively [6], [9], the observed tellurite/cefotaxime synergistic effect would be most probably due to an oxidative stress outbreak. This idea is reinforced even with sub lethal antibiotic concentrations, where enhanced DNA damage and mutation rate are observed [32]. Experiments to address this issue are currently being carried out in our laboratory.

The idea of using TeO_3_^2−^ lies on its extremely high toxicity to bacteria as compared to other metals or non metals as chromium, lead, or manganese [14]. In addition to establishing an oxidative stress status (9), the existence of multiple tellurite cell targets (13, 14) makes the emergence of strains resistant to the antibiotic-potentiating strategy is almost negligible.

Our findings strongly suggest that the use of tellurite (or similar antimicrobials) as an antibiotic-potentiating adjuvant is a novel and feasible strategy to face the antibacterial multi-resistance problem. It is also particularly promising given that the antibacterial-potentiating effect was observed with antibiotic-resistant clinical isolates.

Finally, unveiling the molecular mechanism of the antibiotic-potentiating effect described in this work should contribute to the development of new molecules or compounds to be applied in new therapies to treat infections caused by antibiotic-resistant bacteria.

## Materials and Methods

### Bacterial strains and culture conditions

*E. coli* BW25113, *P. aeruginosa* PAO1 and *S. aureus* were routinely grown in Luria Bertani broth at 37°C with shaking. Minimal inhibitory concentrations (MIC) were determined by serial dilutions as described [11]. Growth inhibition zones were determined as reported previously [9]. Briefly, cells were spread on LB plates amended with TeO_3_^2−^ (0.2, 4 and 200 µM for *E. coli*, *P. aeruginosa* and *S. aureus*, respectively). Sterile filter paper disks (6 mm) containing ampicillin (100 µg, AMP), tetracycline (30 µg, TET), cefotaxime (60 µg, CTX), chloramphenicol (25 µg, CHL) or gentamicin (10 µg, GEN) were placed on the plate centres and incubated overnight at 37°C.

### Antibiotic susceptibility of clinical isolates

Twenty uropathogenic *E. coli* were isolated from patients displaying urinary infection and purified by streaking on MacConkey and LB agar plates. Identification was carried out by conventional microbiological procedures. Cells were grown overnight in LB media (OD_600_∼0.6) and 50 µl were plated on Müeller Hinton plates that contained or not 200 or 400 nM tellurite. Sensidisks containing cefotaxime (CTX, 30 µg), cefalotin (CEFL, 30 µg), ampicillin (AMP, 10 µg), neomycin (NEO, 30 µg), streptomycin (STR, 10 µg), gentamycin (GEN, 10 µg), amikacin (AMK, 30 µg), kanamycin (KAN, 30 µg) or tobramycin (TOB, 10 µg) were used in disk diffusion assays as described above. Growth inhibition zones were determined for each antibiotic in the absence or presence of 200 or 400 nM tellurite for all 20 isolates and averaged. Results were expressed as the mean of 3 independent trials.

### Ethics statement

An individual, informed, written consent was obtained from each participant allowing the samples to be used in this study. Procedures for handling clinical samples were carried out in as recommended in Biosafety Laboratory Manual (3^rd^ Ed., WHO, 2005). Microbiological procedures as well as carrying out this study were specifically approved by the Ethics Committee of Facultad de Ciencias, Universidad San Luis Gonzaga, Ica, Perú.

### Viability assays

*E. coli* grown to OD_600_∼0.3 was exposed to tellurite (0.05 µg/ml) alone or in combination with CTX (0.065, 0.13 or 0.5 µg/ml). At different time intervals samples were serially diluted (1∶10) in PBS buffer, pH 7.0. Ten µl of each dilution were seeded on LB plates to determine the number of colony-forming units (CFU). The CFU/ml was determined using the formula: [(#colonies)*(dilution factor)]/(volume plated in ml) as previously described [6].

### Growth curves

*E. coli* grown to OD_600_∼0.4 was incubated in the presence of CTX or tellurite (see above for concentrations) at 37°C with shaking and absorbance was recorded at 600 nm.
